# Supplementary material for: Large-scale deorphanization of Nematostella vectensis neuropeptide G protein-coupled receptors supports the independent expansion of bilaterian and cnidarian peptidergic systems
Source: eLife. 2024 May 10;12:RP90674. doi: 10.7554/eLife.90674 (PMC11087051; doi:10.7554/eLife.90674)
Supplement: Supplementary file 12. — The file contains the detailed dotplots of Figure 5 with cell-type resolution of the developmental and the adult single-cell dataset. [file elife-90674-supp12.pdf]

A

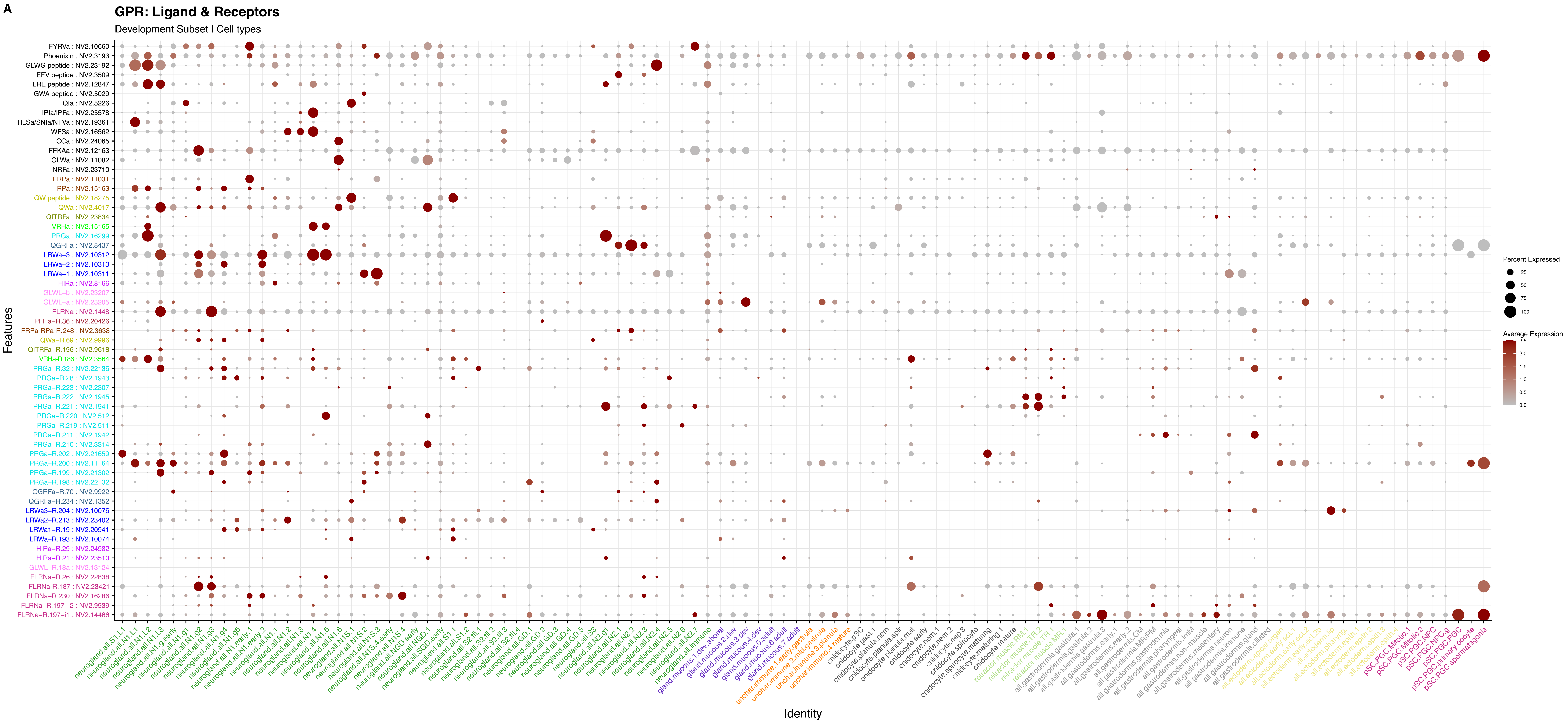
GPR: Ligand & Receptors
Development Subset I Cell types
FYRVa : NV2.10660
Phoenixin : NV2.3193
GLWG peptide : NV2.23192
EFV peptide : NV2.3509
LRE peptide : NV2.12847
GWA peptide : NV2.5029
QIa : NV2.5226
IPIa/IPFa : NV2.25578
HLSa/SNIa/NTVa : NV2.19361
WFSa : NV2.16562
CCa : NV2.24065
FFKAa : NV2.12163
GLWa : NV2.11082
NRFa : NV2.23710
FRPa : NV2.11031
RPa : NV2.15163
QW peptide : NV2.18275
QWa : NV2.4017
QITRFa : NV2.23834
VRHa : NV2.15165
PRGa : NV2.16299
QGRFa : NV2.8437
LRWa−3 : NV2.10312
LRWa−2 : NV2.10313
LRWa−1 : NV2.10311
HIRa : NV2.8166
GLWL−b : NV2.23207
GLWL−a : NV2.23205
FLRNa : NV2.1448
PFHa−R.36 : NV2.20426
FRPa-RPa-R.248 : NV2.3638
QWa−R.69 : NV2.9996
QITRFa−R.196 : NV2.9618
VRHa−R.166 : NV2.3564
PRGa−R.32 : NV2.22136
PRGa−R.28 : NV2.1943
PRGa−R.223 : NV2.2307
PRGa−R.222 : NV2.1945
PRGa−R.221 : NV2.1941
PRGa−R.220 : NV2.512
PRGa−R.219 : NV2.511
PRGa−R.211 : NV2.1942
PRGa−R.210 : NV2.3314
PRGa−R.202 : NV2.21659
PRGa−R.200 : NV2.11164
PRGa−R.199 : NV2.21302
PRGa−R.198 : NV2.22132
QGRFa−R.70 : NV2.9922
QGRFa−R.234 : NV2.1352
LRWa3−R.204 : NV2.10076
LRWa2−R.213 : NV2.23402
LRWa1−R.19 : NV2.20941
LRWa−R.193 : NV2.10074
HIRa−R.29 : NV2.24982
HIRa−R.21 : NV2.23510
GLWL−R.18a : NV2.13124
FLRNa−R.26 : NV2.22838
FLRNa−R.187 : NV2.23421
FLRNa−R.230 : NV2.16286
FLRNa−R.197−i2 : NV2.9939
FLRNa−R.197−i1 : NV2.14466
Features
Identity
Percent Expressed
25
50
75
100
Average Expression
2.5
2.0
1.5
1.0
0.5
0.0

B

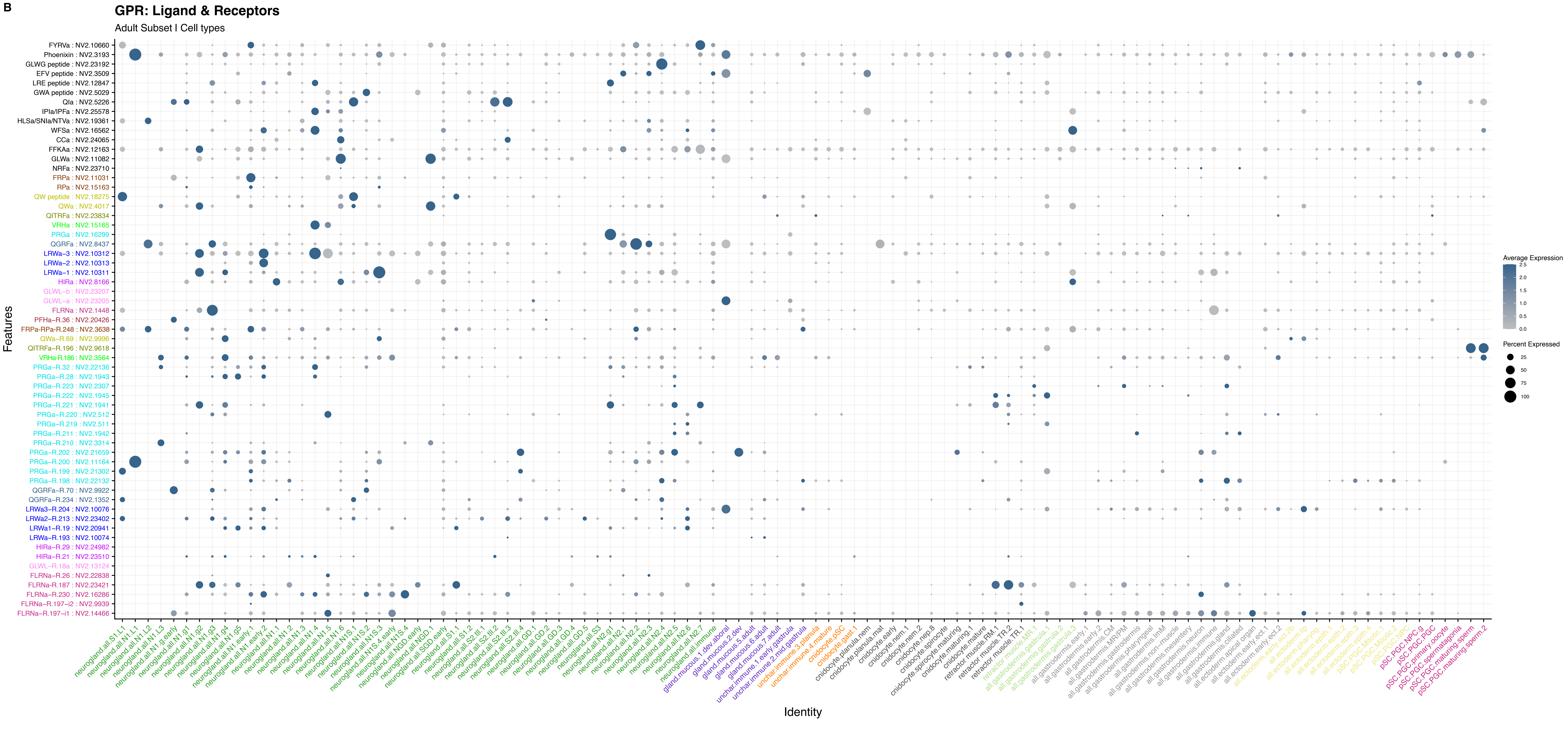
GPR: Ligand & Receptors
Adult Subset I Cell types
FYRVa : NV2.10660
Phoenixin : NV2.3193
GLWG peptide : NV2.23192
EFV peptide : NV2.3509
LRE peptide : NV2.12847
GWA peptide : NV2.5029
QIa : NV2.5226
IPIa/IPFa : NV2.25578
HLSa/SNIa/NTVa : NV2.19361
WFSa : NV2.16562
CCa : NV2.24065
FFKAa : NV2.12163
GLWa : NV2.11082
NRFa : NV2.23710
FRPa : NV2.11031
RPa : NV2.15163
QW peptide : NV2.18275
QWa : NV2.4017
QITRFa : NV2.23834
VRHa : NV2.15165
PRGa : NV2.16299
QGRFa : NV2.8437
LRWa−3 : NV2.10312
LRWa−2 : NV2.10313
LRWa−1 : NV2.10311
HIRa : NV2.8166
GLWL−b : NV2.23207
GLWL−a : NV2.23205
FLRNa : NV2.1448
PFHa−R.36 : NV2.20426
FRPa-RPa-R.248 : NV2.3638
QWa−R.69 : NV2.9996
QITRFa−R.196 : NV2.9618
VRHa−R.166 : NV2.3564
PRGa−R.32 : NV2.22136
PRGa−R.28 : NV2.1943
PRGa−R.223 : NV2.2307
PRGa−R.222 : NV2.1945
PRGa−R.221 : NV2.1941
PRGa−R.220 : NV2.512
PRGa−R.219 : NV2.511
PRGa−R.211 : NV2.1942
PRGa−R.210 : NV2.3314
PRGa−R.202 : NV2.21659
PRGa−R.200 : NV2.11164
PRGa−R.199 : NV2.21302
PRGa−R.198 : NV2.22132
QGRFa−R.70 : NV2.9922
QGRFa−R.234 : NV2.1352
LRWa3−R.204 : NV2.10076
LRWa2−R.213 : NV2.23402
LRWa1−R.19 : NV2.20941
LRWa−R.193 : NV2.10074
HIRa−R.29 : NV2.24982
HIRa−R.21 : NV2.23510
GLWL−R.18a : NV2.13124
FLRNa−R.26 : NV2.22838
FLRNa−R.187 : NV2.23421
FLRNa−R.230 : NV2.16286
FLRNa−R.197−i2 : NV2.9939
FLRNa−R.197−i1 : NV2.14466
Features
Identity
Average Expression
2.5
2.0
1.5
1.0
0.5
0.0
Percent Expressed
25
50
75
100
