# Supplementary material for: Large-scale deorphanization of Nematostella vectensis neuropeptide G protein-coupled receptors supports the independent expansion of bilaterian and cnidarian peptidergic systems
Source: eLife. 2024 May 10;12:RP90674. doi: 10.7554/eLife.90674 (PMC11087051; doi:10.7554/eLife.90674)
Supplement: Supplementary file 11. — The file contains the detailed trees that are shown in Figure 4 and Figure 4—figure supplements 1 and 2, with exact support values and sequence identifiers of all branches. [file elife-90674-supp11.pdf]

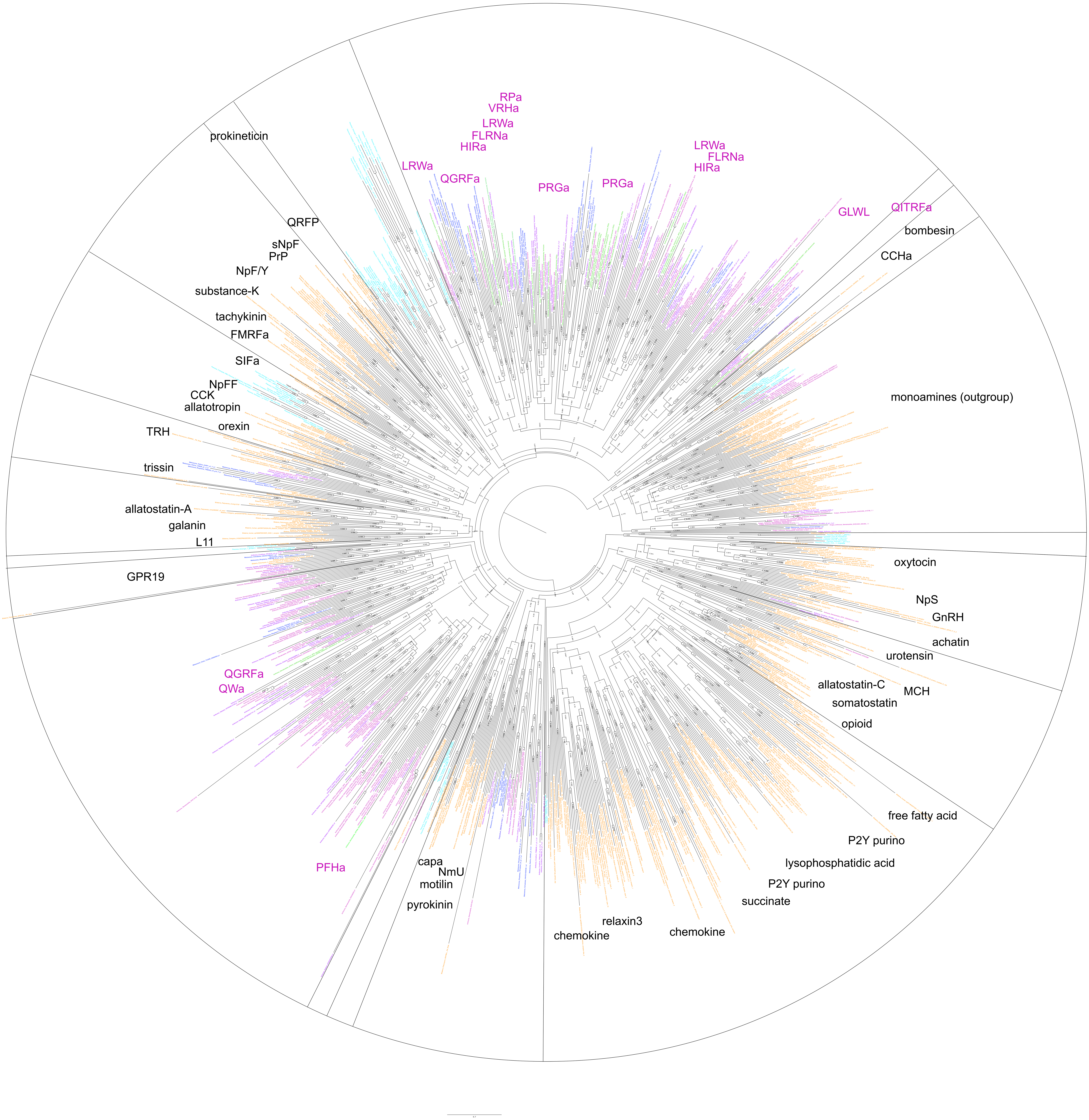

prokineticin
RPa
VRHa
LRWa
FLRNa
HIRa
LRWa
QGRFa
PRGa
PRGa
LRWa
FLRNa
HIRa
GLWL
QITRFa
bombesin
CCHa
QRFP
sNpF
PrP
NpF/Y
substance-K
tachykinin
FMRFa
SIFa
NpFF
CCK
allatotropin
orexin
TRH
trissin
allatostatin-A
galanin
L11
GPR19
monoamines (outgroup)
oxytocin
NpS
GnRH
achatin
urotensin
allatostatin-C
MCH
somatostatin
opioid
QGRFa
QWa
free fatty acid
P2Y purino
lysophosphatidic acid
P2Y purino
succinate
PFHa
capa
NmU
motilin
pyrokinin
relaxin3
chemokine
chemokine

Supplementary tree1: Tree (FastTree) of neuropeptide GPCRs and related bilaterian chemokine receptors. Bilaterian sequences are shown in yellow, cnidarian sequences are shown in dark blue (Medusozoa) and magenta (Anthozoa), placozoan sequences are shown in light blue. Deorphanized Nematostella vectensis sequences are shown in green. Sequences were aligned using muscle, alignment was trimmed with the gappyout option of trimal, tree was calculated using FastTree. Abbreviations: a = amide, CCK = cholecystokinin, GnRH = gonadotropn releasing hormone, GPR19 = G protein-coupled receptor 19, L11 = elevenin, MCH = melanin concentrating hormone, Nm-U = neuromedin U, NpFF = neuropeptide FF, NpS = neuropeptide S, NpY/F = neuropeptide Y/neuropeptide F, PrP = prolactin releasing peptide, sNpF = short neuropeptide F, TRH = thyrotropin releasing hormone,

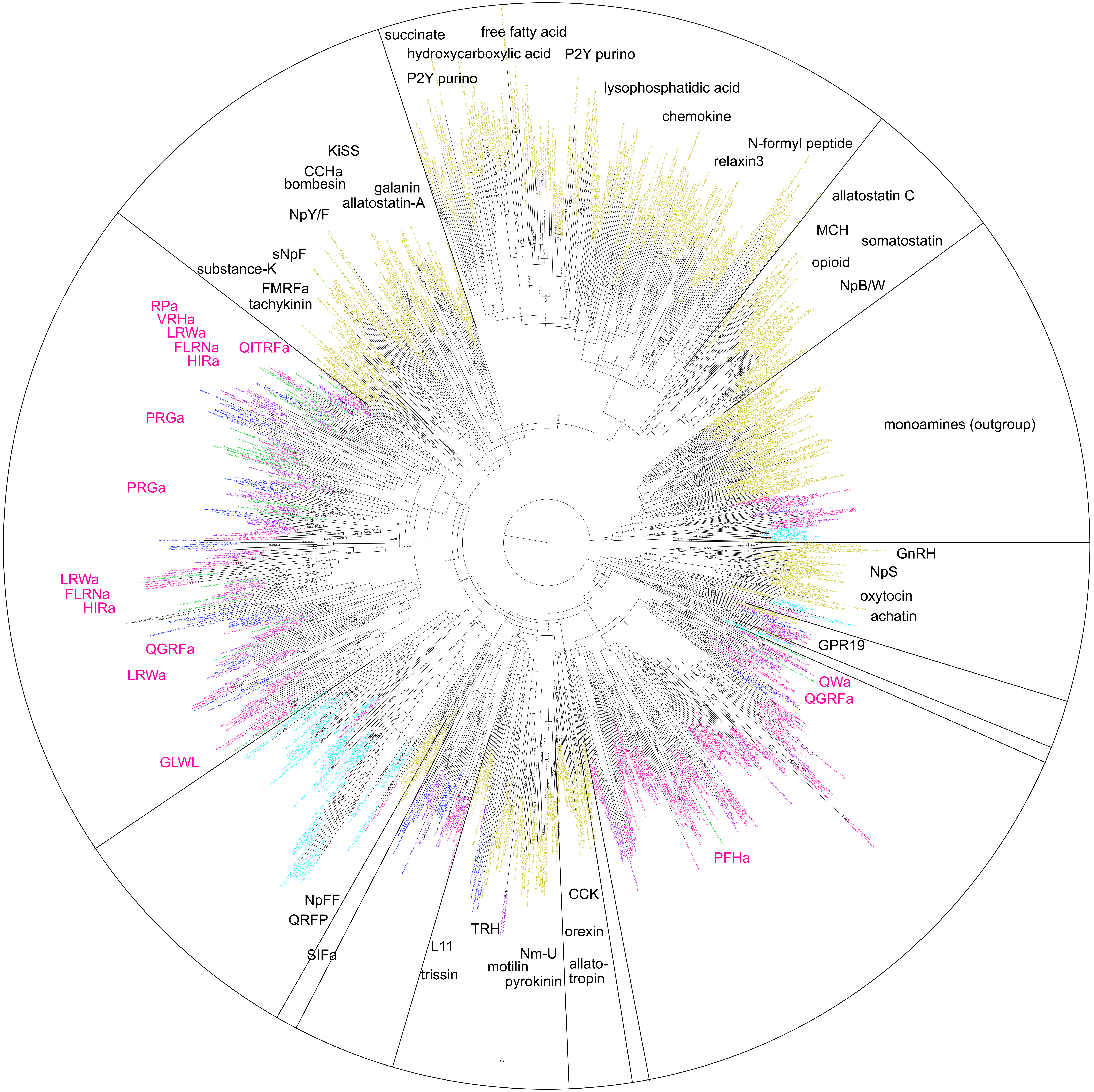
succinate
free fatty acid
hydroxycarboxylic acid
P2Y purino
P2Y purino
lysophosphatidic acid
chemokine
N-formyl peptide
relaxin3
allatostatin C
MCH
somatostatin
opioid
NpB/W
monoamines (outgroup)
GnRH
NpS
oxytocin
achatin
GPR19
QWa
QGRFa
PFHa
CCK
orexin
allato-
tropin
Nm-U
motilin
pyrokinin
TRH
L11
trissin
NpFF
QRFP
SIFa
GLWL
LRWa
QGRFa
LRWa
FLRNa
HIRa
PRGa
PRGa
RPa
VRHa
LRWa
FLRNa
HIRa
QITRFa
FMRFa
tachykinin
substance-K
sNpF
NpY/F
KiSS
CCHa
bombesin
galanin
allatostatin-A

Supplementary tree2: Tree (IQtree) of neuropeptide GPCRs and related bilaterian chemokine receptors. Bilaterian sequences are shown in yellow, cnidarian sequences are shown in dark blue (Medusozoa) and magenta (Anthozoa), placozoan sequences are shown in light blue. Deorphanized Nematostella vectensis sequences are shown in green. Sequences were aligned using mafft, alignment was trimmed with the gappyout option of trimal, tree was calculated using IQtree. Abbreviations: a = amide, CCK = cholecystokinin, GnRH = gonadotropn releasing hormone, GPR19 = G protein-coupled receptor 19, L11 = elevenin, MCH = melanin concentrating hormone, Nm-U = neuromedin U, NpB/W = neuropeptide B/neuropeptide W, NpFF = neuropeptide FF, NpS = neuropeptide S, NpY/F = neuropeptide Y/neuropeptide F, sNpF = short neuropeptide F,

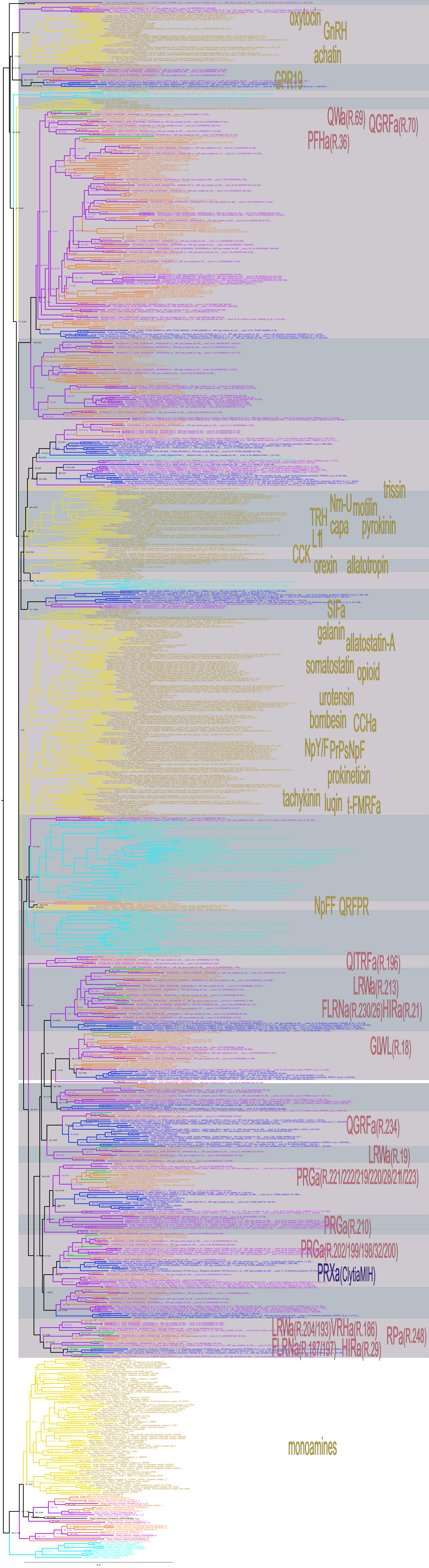

oxytocin
GnRH
achatin
GPR19
QWa(R.69)
QGRFa(R.70)
PFHa(R.36)
trissin
Nm-U
motilin
TRH
capa
pyrokinin
CCK
orexin
allatotropin
SIFa
galanin
allatostatin-A
somatostatin
opioid
urotensin
bombesin
CCHa
NpY/F
PrP
sNpF
prokineticin
tachykinin
luqin
t-FMRFa
NpFF
QRFPR
QITRFa(R.196)
LRWa(R.213)
FLRNa(R.230/26)
HIRa(R.21)
GLWL(R.18)
QGRFa(R.234)
LRWa(R.19)
PRGa(R.221/222/219/220/28/211/223)
PRGa(R.210)
PRGa(R.202/199/198/32/200)
PRXa(ClytiaMIH)
LRWa(R.204/193)
VRHa(R.186)
RPa(R.248)
FLRNa(R.187/197)
HIRa(R.29)
monoamines

Supplementary tree 3: Phylogeny of metazoan class A neuropeptide GPCRs. Same tree as figure 4 in the main manuscript, but with added branch annotations and exact support values (aBayes/aLRT-SH-like support). Bilaterian sequences are shown in yellow, cnidarian sequences are shown in dark blue (medusozoan species) and magenta (Aiptasia and Corallium) and orange (Nematostella), placozoan sequences are shown in light blue. Branches of deorphanized Nematostella GPCRs are shown in green. Alternating shades behind the tree branches highlight different monophyletic groups. Abbreviations: a = amide, B = Bilateria, CCK = cholecystokinin, GnRH = gonadotropin releasing hormone, MIH = maturation-inducing hormone, Nm-U = neuromedin U, NpFF = neuropeptide FF, NpY/F = neuropeptide Y/neuropeptide F, P = Placozoa, PrP = prolactin releasing peptide, R.# = Nematostella GPCR number, sNpF = short neuropeptide F, t-FMRFa = trochozoan FMRFamide, TRH = thyrotropin releasing hormone.
