# Supplementary material for: Large-scale deorphanization of Nematostella vectensis neuropeptide G protein-coupled receptors supports the independent expansion of bilaterian and cnidarian peptidergic systems
Source: eLife. 2024 May 10;12:RP90674. doi: 10.7554/eLife.90674 (PMC11087051; doi:10.7554/eLife.90674)
Supplement: Supplementary file 57 [file elife-90674-supp2.zip › Supplementary_file_2.odt]

33 propeptides that encode 30 different peptide groups [FLRNamide, LRWamide1 & QITRFamide seem to have 2 different precursors (potential paralogs) that encode the same peptides]

66 peptides were tested

Blue: Signal peptide

purple: dibasic cleavage site

red: amidation and cleavage site

yellow: tested peptides

green: cysteine residues

Bold: detected by MS (Hayakawa et al. 2019)

Underlined: detected by MS (this study)

________________________________________________________________________________________________________________________________________________________________________

------------

GLWL peptide

------------

>Nvec EDO30284.p1

>NVE20692

MKSFIPTILALIAAMSSVMPVPIKQGNTKEIIEPGQRSQNGHCPPGLWLCKANTRLSRQNRHCPPGLWLCKANTRRRRQNGHCPPGLWLCKANTRLRRQNGHCPPGLWLCKANTRRRRQNGHCPPGLWLCKANTRLRRQNGHCPPGLWLCKANTRLRRQNGHCPPGLWLCKANTRLRRQNGHCPPGLWLCKANTRLIRQNGHCPPGLWLCKANTRLRRQNGHCPPGLWLCKANSRLRRQNEHCPPGLWLCKANSRLRRQNGHCPPGLWLCKANTRLRRQNGHCPPGLWLCKANTRLRRQNGHCPPGLWLCKANTRLRRQNGHCPPGLWLCKANSRLRRQNEHCPPGLWLCKANTRLRRQNGHCPPGLWLCKANTRLRRQNGHCPPGLWLCKANSRLRRQNGHCPPGLWLCKANTRLRRQNGHCPPGLWRCKANSRLRRQNGHCPPGLWLCKANSRLRRQNGHCPPGLWLCKANTRLRRQNRHCPPGLWLCKANTRLRRQNGHCPPGLWLCKTNTRLRRQNGHCPPGLWLCKANIEH*

>Nvec EDO42328.p1

MKSFIPTILALIAAMSSVMPVPVKQGNTKEIIEPGQRSQNGHCPPGLWLCKSNSRLRRQNGHCPPGLWLCKANTRQRRQNGHCPPGLWLCKANSRLRRQNGHCPPGLWLCKANTRLRRQNGHCPPGLWLCKANTRLRRQNGHCPPGLWLCKANTRLRRQNGHCPPGLWLCKANTRLRRQNGHCPPGLWLCKANTRLRRQNGHCPPGLWLCKANTRLRRQNGHCPPGLWLCKANTRLRRQNGHCPPGLWLCKANTRQRRQNGHCPPGLWLKLQKLEKSYKGQTNAKRRQNRKTIPISNTISHLRMKSFIPTILALIAAMSSVMPVPVKQGNTKEIIEPGQRSQNGHCPPGLWLCKANSRLRRQNGHCPPGLWLCKANTRLRRQNGHCPPGLWLCKANTRLRRQNGHCPPGLWLCKANTRLRRQNGHCLPWLWLCKANTRLKRQNGHCPPGLWLCKANTRLRRQNGHCPPGLWLCKANTRLRRQNGHCPPGLWLCKANSRLRRQNGHCPPGLWLCKANTRQRRQNGHCPPGLWLCKANTRQRRQNGHCPPGLWLCKANTRQRRQNGHCPPGLWLCKANTRRRRQNGHCPPGLWLCKANTRRRRQNGHCPLGLWLCKANTRLRRQNGHCPPGLWLCKANTRLRRQNGHCPPGLWLCKANTRLRRQNGRCPPGLWLCKANTRLRRQNGHCPPGLWLCKANTRLRRQNGHCPPGLWLCKANSRLRRQNGHCPPGLWLCKANTRLKRQNGHCPPGLRLNIRLKQKAPTTR*

>NVE20691

MKSFIPTILAMIAAMSRVMPAPVKQGNTKEIIEPGQRRQNGHCPPGLWLCKANSRLRRQNGHCPPGLWLCKANSRLRRQNGHCPPGLWLCKANTRLRRQNGHCPPGLWLCKANSRLRRQNGQCPPGLWLCKANSRLRRQNGHCPPGLWLCKANSRLRRQNGHCPPGLWLCKANSRLRRQNGHCPPGLWLCKANSRLRRQNGHCPPGLWLCKANSRLRRQNGHCPPGLWLCKANSRQRRQNGHCPPGLWLCKANSRLRRQNGHCPPGLWLCKANSRLRRQNGHCPPGLWFCKANTRLRRQNGHCPPGLWLCKAKTRLRRQNGHCPPGLWLCKAKTRLRRQNGHCPPGLWLCKANIEH

tested peptides (round1):

GLWL: QNGHCPPGLWLCKANTRL

GLWLmdm: CPPGLWLCKANTRL

GLWLshrt: CPPGLWLC

GLWLcc: QNGHCPPGLWLCKANTRL (5-12 cysteine bridge)

activates receptor:

GPR18, GPR18a, GPR18b

.

...

.....

________________________________________________________________________________________________________________________________________________________________________

---------

FLRNamide

---------

>HADO01000428.1 NVE17328 antho-RNamide1 (Koch & Grimmelikhuijzen 2020)

>NVE17328

MASSRTLVAFLAVFVLCGMLIQTSDAFLRNGRDEIYAKKRAEMGYPPRWEEYENQLYQRRDEDAKRSKQ*

>HADP01211238.1 antho-RNamide2 (Koch & Grimmelikhuijzen 2020)

MKRSLVVVLVVAIAAAMFVETEGFLRNGREQIPSKLERDAAELEDFYNALYAQQRANPGYDRFRRERKANA*

tested peptides (round1.5):

FLRNa: FLRN-amide

activates receptor:

GPR26

GPR187

GPR197

GPR230

.

...

.....

____________________________________________________________________________________________________________________________________________________________________

----------

QITRFamide

----------

>scaffold_47 pQITRFamide1 (Koch & Grimmelikhuijzen 2020)

>NVE20859

>NVE14778

MTYKSLLHVFIFLLSTCSAYGRSTRVSLKTTVTGGITRQPIPLRSTGYPSGSAYHSASLPIVRFTRSASPQITRFRKSDASPQITRFGRSASPQKTRFGRSASPQITRFGRSASPQITRFGRSASPQITRFGRSASPQITRFGRSASPQITRFEVGRLITRFGSHFQ

>HADP01261925.1 pQITRFamide2 (Koch & Grimmelikhuijzen 2020)

MPNTCLIHVSLIVILTLYGATKGRGTPASRNTIVSRGIITASSNTFKGGIHRSKAVKSSPRNGVSTIASSEITSVRKSLKLPHIKELIKFASQPVARFGRSASPQITRFGRSASPQITRFGRSAS

tested peptides (round1):

RFa0: pyro-QITRFamide

activates receptor:

GPR70 (at higher concentrations than QGRFamide)

GPR196

.

...

.....

________________________________________________________________________________________________________________________________________________________________________

-------

RFamide

-------

>Nvec RFamide (RFa, Hayakawa et al. 2019, 2022)  (pQGRFamide, Koch & Grimmelikhuijzen 2020)

>NVEC200_014078_1.1

>NVE7549

MAPRPGTLLLLGILIQVLICTAKSTYKKEIADLLDDNKDTPQFWKGRFSDPQFWKGRFSDPQFWKGRFSDPQFWKGRFSDPQFWKGRFSDPQFWKGRFSDPQFWKGRFSDPQFWKGRFADELLNGGHHKHHHEEGEWKRTAGPGRFGREDQGRFGREDQGRFGREDQGRFGREDQGRFGREDQGRFGREDQGRFGREDQGRFGREDQGRFGREDQGRFGREDQGRFGREDQGRFGREDQGRFGREDQGRFGREDQGRFGREDQGRFGREDQGRFGREDQGRFGREDQGRFGREDQGRFGREDQGRFGREDQGRFGREDQGRFGRNKIARVIDLDQGRFGRTMDTATKKDTVASMPEQDANPQTRFDGKKRQAAKERSIEKKSTISSDAKASDAKQS

tested peptides (round1):

QGRFa: pyro-QGRF-amide

RFa1: QGRFGREDQGRF-amide

RFa2: MPEQDANPQTRFD-amide

QGRFa activates receptor:

GPR70

GPR234

.

...

.....

________________________________________________________________________________________________________________________________________________________________________

---------

LRWamide1

---------

>HADP01080760.1 antho-RWamide2 (Koch & Grimmelikhuijzen 2020)

>NVE24307

MESKRLLIVLVVCCALVSLYVEPSQAQGLRWGREFEEEHPKLSPLMKEYLRRQEAMQKKREFAAKKADY*

>HADP01056499.1 NVE11178 antho-RWamide3 (Koch & Grimmelikhuijzen 2020)

>NVE11178

MESKRLLVILVVCFALVAFAPSTEAQGLRWGREYDESPEKLVPMMKEYIRRQEELKKKREFSMN*

tested peptides (round1):

LRWa1: pyro-QGLRW-amide

activates receptor:

GPR19

GPR193

GPR204

GPR213

.

...

.....

________________________________________________________________________________________________________________________________________________________________________

---------

LRWamide2

---------

>HADP01189706.1 antho-RWamide4 (Koch & Grimmelikhuijzen 2020)

>NVE24305

MDAKKVLLIAVISCVLLSVCIEQTTAQGGLRWGREFENEFQQWKDAHYPSREERRSFQKKRSFKMA*

tested peptides (round1):

LRWa2: pyro-QGGLRW-amide

activates receptor:

GPR19

GPR193

GPR204

GPR213

.

...

.....

________________________________________________________________________________________________________________________________________________________________________

---------

LRWamide3

---------

>HADP01262889.1 NVE2302 antho-RWamide1 (Koch & Grimmelikhuijzen 2020) (also 5’partial: NVE24308) (Hayakawa et al 2022)

>NVE24308

MASKTLLVCLLVTFMVLSIYTQESSAGPPQGLRWGKRWENPSEKQVRENAEREVQDFKDYFKKKYNRDLDI*

tested peptides (round1):

LRWa3: GPPQGLRW-amide

activates receptor:

GPR19

GPR193

GPR204

GPR213

.

...

.....

________________________________________________________________________________________________________________________________________________________________________

--------

HIRamide

--------

>Nvec NVE15069 HIRamide (Hayakawa et al. 2019, 2022) (FHIRamide, Koch & Grimmelikhuijzen 2020) (NVEC200_013206_1.1) (NVE15069)

>NVEC200_013206_1.1

MRLYLFVPVFALVLAVEGASDEKRDSKQPPIDLSPAAYFHIRGKRTYNAPPLDLSGPAYFHIRGKRTAKQPPYLDLGEPSFFHIRGKRTEGPPYIDLTEPSFFHIRGKRSSEQPPLDLGPAYFHIRGKRTKNPPIDLGPAYFHIRGKRLSGEQPPYLDLTPAYFHIRGKRTQQPPMIDLSEPAFFHIRGRRAVEQPPYLDLTPSYFHIRGKRTEYPPFLELGQPSYFHIRGRRAEKTTKD

tested peptides (round1):

HIRa1: pyro-QPPYLDLGEPSFFHIR-amide

HIRa2: YPPFLELGQPSYFHIR-amide

HIRa3: pyro-QPPYLDLTPAYFHIR-amide

HIRa4: pyro-QPPLDLGPAYFHIR-amide

HIRa5: NPPIDLGPAYFHIR-amide

activates receptor:

GPR21, GPR29

.

...

.....

________________________________________________________________________________________________________________________________________________________________________

--------

PRGamide

--------

>Nvec NVE226 PRGamide (Hayakawa et al. 2019, 2022) (XPRXamide, Koch & Grimmelikhuijzen 2020)

MAYFKWTLCAFVLAVLCLSTNYVAGEKNEEKSEKSTLLQELSKKDFDKELAADEAIVLELLKEKADDLDEESLLDLLAPRGGRDAPRGGRSLLDAPRGGRSLLDLLAAPRGGRDAPRGGRSLTELLNAPRRGRSLADLLDAPRGGRSLAELLDAPRGGRSIEAPRGGRSLAELLEAPRGGRSLIELLEAPRGGRSVEAPRGGRSILELLTAPRGGRSAPRGGRSAEKSDAIHKEKKAPRGGRKRRSLPEEEMDGPRGGRSAVSGRSSEDGPRGGRAVYGPRGGRSFDGSRGGRSYDGPRGGRSMENGPRGGRATEFGPRGGRSYEGSRGGREIDGPRGGRSFKEGPRGGRAVEGPRGGRDLYEEGPRGGREVYLEGPRGGRDLVLEGPRGGRDLYEEGPRGGREVNLEGPRGGRDLYEEGPRGGRSYDGPRGGRSAVYGPRGGRDANDGPRGGRDANDGPRGGRDANDGPRGGRDANEGPRGGRDANEGPRGGRDANKGPRGGRNTNTVGAQQADARQGQRGGRSTGMGPRGGRSVSSEKDNRH

tested peptides (round1):

PRGa: GPRG-amide

PRGa1: APRGGRSLLDLLAAPRG-amide

PRGa2: GPRGGREVNLEGPRG-amide

PRGa3: GPRGGRATEFGPRG-amide

PRGa4: GPRGGRSYDGPRG-amide

PRGa5: APRGGRDAPRG-amide

activates receptor:

GPR28, 

GPR32, 

GPR198, 

GPR199, 

GPR200, 

GPR202, 

GPR210, 

GPR211, 

GPR219, 

GPR220, 

GPR221, 

GPR222, 

GPR223

.

...

.....

________________________________________________________________________________________________________________________________________________________________________

--------

PFHamide

--------

>NVE22506 (predicted extracellular, soluble with DeepLoc1.0)

MADIPALIPVPVPYGGSLLKSGSLGLSSDDLHTALYTGLVGFKTRYFTTVAMETCTPLTSVVYHTSEKDGLEVTTTNYYGIVLEEREPEIFKLPEYCTDTRKRRQMPNMVPFHGKRQMPNMVPFHGKRQMPNMVPFHGKRQMPNMVPFHGKRQMPNQVPYLG

tested peptides (round2):

PFHa: pyro-QMPNMVPFH-amide

activates receptor:

GPR36

.

...

.....

________________________________________________________________________________________________________________________________________________________________________

-------

QWamide

-------

>Nvec QWamide (NQWa, Hayakawa et al. 2019, 2022) (NVE4601)

MLKLITLAKSAQFKLTKRSRIYIKASFYNHAWCLHRLLWLSSYCRKSKFRQGLTDQPMDESKEIPPQGLRFSQWGKKEIPPQGFRFNQWGKKEIPPQGLRFNQWGKKEIPPQGFRFSQWGKKEIPPQGFRFNQWGKKEIPPQGLRFNQWGKKEIPPQGLRFNQWGKKEIPPQGLRFSQWGKREIPPQGLRFNQWGKKEIPPQGLRFNQWGKKEIPPQGLRFNQWGKKEIPPQGLRFNQWGKKEIPPQGLRFNQWGKKEIPPQGLRFNQWGKKEIPPQGLRFSQWGKKEIPPQGLRFNQWGKKEIPPQGLRFNQWGKRKLINQVMI

>NVEC200_009895_1.1 protein AED:0.20 eAED:1.00 QI:0|-1|0|1|-1|1|1|0|294

MRGVFIVFFGFLLTVVSAKSAEERPTKDVRREIPPQGFRFNQWGKKEIPPQGFRFNQWGKKEIPPQGLRFNQWGKKEIPPQGFRFNQWGKKEIPPQGFRFNQWGKKEIPPQGFRFNQWGKKEIPPQGLRFNQWGKKEIPPQGLRFSQWGKKEIPPQGFRFNQWEKKEIPPQGLRFNQWGKKEIPPQGLRFNQWGKKEIPPQGLRFSQWGKREIPPQGLRFNQWGKKEIPPQGLRFNQWGKKEIPPQGLRFSQWGKKEIPPQGLRFNQWGKKEIPPQGLRFNQWGKRKLINQVMI

tested peptides (round1):

QWa1: IPPQGFRFSQW-amide

QWa2: IPPQGLRFNQW-amide

activates receptor:

GPR69

.

...

..…

________________________________________________________________________________________________________________________________________________________________________

--------

VRHamide

--------

>Nvec NVE12742 VRHamide (VRHa, Hayakawa et al. 2019, 2022)

>NVEC200_002800_1.1 

>NVE12742 partial C-terminus

MKITPVMSCLVLASALLVTAECYRITDPGDLEKPQENDEPGPPMIKIPVRHGKREFNDDLEDTTLLHYPFRVARKRELFNAGGPPPIYLPVRPGKRGYYRDNIQETRQYLDPRPGKRSRNH

tested peptides (round1):

RHa1: GPPMIKIPVRH-amide

RHa2: GPPMIKIPVRH-amide (oxidation of methionine)

RHa3: GPPPIYLPVRP-amide

RHa4: GYYRDNIQETRQYLDPRP-amide

RHa5: pyro-QYLDPRP-amide

activates receptor:

GPR186 (RHa1)

.

...

.....

________________________________________________________________________________________________________________________________________________________________________

-------

HRPamide

-------

>Nvec RPamide_EDO37503.p1 (PRPa, Hayakawa et al. 2019, 2022)

>NVEC200_002806_1.1 

>similar to NVE3755 (NV_RPamide III in Gilbert et al 2022)

MKVLCVTLVLMALVCSVQSRNLRHKDYERELQDYRRDFARALSMVEQDAFLPKPRPGRREQDSSNYEFPPGFHRPGKKRSFKEEK

>NVE3755

MKVLCVTLVLMALVCSVQSRNLRHKDYERELQDYRRDFARALSMVEQDAFLPKPRPGRREFPPGFHRPGKKRSFKEEK

tested peptides (round1):

RPa1: pyro-QDAFLPKPRP-amide

RPa2: FPPGFHRP-amide

activates receptor:

GPR248

.

...

....

________________________________________________________________________________________________________________________________________________________________________

--------

FRPamide

--------

>Nvec XP_032233649.1 FRPamide (antho-RPamide, Koch & Grimmelikhuijzen 2020)(FRPa, Hayakawa 2022)

>NVE4012

MYSCMGISLLLILCFKGSYGEESIDLEPGVDKVPEKVEHGEEGSAKYNINTVTLTGNKATGNKVTNDDKRPPWPPRPGKRTFRPQESADTPSIFRPGRSVHEDQLLFRPGRANLHKRQGLMFRPGRREDVPKPQIFRPGRREDIPSDDDQLMFRPGRNEEQLFRPGRRSDVGDEQLLFRPGRRSDLEEDQLIFRPGRRSDVAEEQLFRPGRRSDVPEAFLDQWTSVRPGRGGYRMPWTYTGNSVNSHVSQKSHTFRQKAVEQEKKKREV

tested peptides (round1):

FRPa1: WPPRP-amide

FRPa2: pyro-QGLMFRP-amide

FRPa3: pyro-QLIFRP-amide

FRPa4: pyro-QLFRP-amide

activates receptor:

GPR248

.

...

.....

________________________________________________________________________________________________________________________________________________________________________

--------

NRFamide

--------

>Nvec EDO34390.p1

>NVE9773 is nearly identical but the short part with the peptides is missing (everything before and after is the same). BLAST suggests this is a collagen, but doesn`t align the part with the peptides. Miss-assembly and NRFamide part is in reality on a different transcript? Not a neuropeptide?) 

MAFRLQAARLFLLVVSFYATLLSFSFAQAQTQNLVCPTVRTSALTQLNCKSPAHNCNSDKECPDGEKCCPLGSQCELLCRKAVKQERCAAAIDLAILLDASEAISPQEWSKLLKFTADLMDYYGISEDGTRISVATFSTDVDIVLSFNEFSGVEMNAASVKRGILGAKQSRGPGLRIDKALKAADKDLFNRRFGMREDQKKVCLLVTSGAQTKDQGPSTQLGTVTALLSARGVDIYAVGVGDGVDSSELRNIASTEDFIYTASSFEEINKVLEPFGTVQCSGCKKPLDLVLGIPTSRTLGDDFKQIKLFFVNLIALFDISPSKTHVGVITFSESAQMSLHMDKMYEPRTVTRFINDITPTGDQMRIDRAIDTAGDYGFTIYGGVRQTMPKTFVLFAPTNSSSSAAANMKAAEKLKSLGVRLLIVGLTADINKVDFSSVVSQPSRKFLFSSAAYDGFTSVRHDMASTLCNACFVPIDVAFIMDNSESVSSEKYDFVKRFVKDVIMSYADAENSANFAIGQYAKYFQTGTKRFRNFRSMEELDEVINSLRQMSSSAERNVGAGLRGAANEFFQVKNGMRQGLPRFLIVLASANPSASSEAIESAVVNLDKENVRRIAVGFTEDATPGFLRMLASDPSLMFRVDEPKKLDKVMMELTPMLCQACKKSMEILFLVESTKFVGEHGFNRVKHFITRLVENFDVSGTTITLAMATYADSAQPVVQFSDSAAWRKAEFQKAVQGALFLGEGRVRTAEALKFADRRIFTSRFGSREDIKKTIVLIGSGRYNEVPTQPYFHTDNSPNRFGRYNETIVLIGSGRYYEVPTQPYFHTDNSPNRFGRYNEVPTQPYFHTDNSPNRFGRYNETIVLIGSGRYNEVPTQPYFHTDNSPNRFGRYNEVPTQPYFHTDNSPNRFGRYNEVPTQPYFHTDNSPNRFGRYNEVPTQPYFHTDNSPNRFGRYNETIVLIGSGRFEEAGLAVAIAEKLYGEGINLVAVAIGNSPDFPALGRLVVNERHEVFPIRSETALEGQRRPLVTAICVGGMKDYIVSRY*

tested peptides (round1):

NRFa: QPYFHTDNSPNRF-amide

activates receptor:

GPR21 (only slightly, and not as strong as HIRamides do)

.

...

.....

________________________________________________________________________________________________________________________________________________________________________

---------

FYRVamide

---------

>HADO01002733.1 antho-RIamide (Koch & Grimmelikhuijzen 2020)

>NVE9956

MSPTMKYVLVAVLFCALVAPRFTHGFYRVGRELEKRREAMGGQDPLVQLAQGPDSDDLWRARMAYLRARRTEAKKTEQR*

tested peptides (round1.5):

FYRVa: FYRV-amide

activates receptor:

GPR197 (but at higher concentrations than FLRNamide)

.

...

.....

________________________________________________________________________________________________________________________________________________________________________

--------

GLWamide

--------

>Nvec GLWamide (MH939200.1 Nematostella vectensis GLWamide precursor) (Koch & Grimmelikhuijzen 2020) (NVEC200_000296_1.1)(GLWa, Hayakawa et al 2022)

>NVE9858

>NVEC200_000296_1.1

MALFGHTLVAVLFCLALCHAETKRKAADTTDTENELDASPNVNNDDDNDIKRMPTDTKRQAGAPGLWGKRDAGPPGLWGKRDAGPPGLWGKRSPKPPGLWGKRQAGAPGLWGKRSAGPPGLWGKRDAGPPGLWGKRVAGPPGLWGKRQAGAPGLWGREAGAPGLWGKRQAGAPGLWGKREAGAPGLWGREANAPGLWGKRRAGAPGLWGKREANAPGLWGKRQAGPPGLWGKREANAPGLWGKRQAGPPGLWGKREANAPGLWGKRQAGPPGLWGKRDEDEDEDMDETNGDPLWGRSADAGPPGLWGRKKRAASPQRDLYGIGLWGRNAALMTAEELDLSFKNEEQS

tested peptides (round1):

GLWa1: QAGAPGLW-amide

GLWa2: QAGPPGLW-amide

GLWa3: GPPGLW-amide

GLWa4: APGLW-amide

GLWa5: SPKPPGLW-amide

.

...

.....

________________________________________________________________________________________________________________________________________________________________________

---------

FFKAamide

---------

>HADO01001257.1 NVE12918 antho-KAamide (Koch & Grimmelikhuijzen 2020)

>NVE12918

MNRLALVLLCMFLTAVLMPNRVQGFFKAGRELKEKSADSNERRQFNEGSVAEEEARKEFLRALLRSAERRSKTDDYQ*

tested peptides (round1.5):

FFKAa: FFKA-amide

.

...

.....

________________________________________________________________________________________________________________________________________________________________________

--------

LRPamide

--------

>Nvec EDO48928.p1 LRPamide

PWSCSLLPGLVAFSLVVWPSPWSCCLRPGRVASPWSCCLRPGRVASCLVLKFSPWSCSLRPGRKVFALVPSPWSCSLRPGRVASPWSYSLRPGRVASPWSCSLRPGRVAFCPGLIPSPWSCSARPGRIAFVLVVLPSPWS

tested peptides (round1):

LRPa1: SPWSCSLRP-amide

LRPa2: SPWSYSLRP-amide

.

...

.....

________________________________________________________________________________________________________________________________________________________________________

-------

CCamide

-------

>NVE15666 (ordered for Acropora millepora: Am_CCamide)

MTAKLCLPLCIYVFCILHCVSTSSLRVKADNNPSDDDALSRFFRRDCHVGLWCGKKREPAIEQSSRFKSADDCPVGLWCGKKRSASKGESIEISHSNKQREQGEEESTVLQKLTDGNTGPPDEVARRMFRRASTSCPPGLWCGKKRSIPEQNPSGLELQLPYLAGTLPSYEVNDEDAPQSDLKKMFRRASTSCPPGLWCGRKRTVYSNIGIPPRQSSLKSKFERSCPPGLWCGK

tested peptides (round2):

Am_CCa: CPPGLWC-amide

.

...

.....

________________________________________________________________________________________________________________________________________________________________________

--------

WFSamide

--------

>NVEC200_005035_1.1 (WFSa, Hayakawa et al 2022)

>NVE2973

>NVE2974

MSNYFLHWVTALAFILCSGLANGKIPKVPIKEVENAPPSTGEGEAGFIPWFSGKREFEPKNALKNDNAPPGNGEGEAGFIPWFSGKREFEPKKALKNDNAPPGNEEGEARFIPWFSGKRELIPKIVLQNDNAPPGNGEGEAGFIPWFSGKREFEQKNALQNDNAPPGNGEGEAGFIPWFSGKREFEPKNALQNDNAPPGNGEGEAGFIPWFSGKRDTTKAAVQNDLVPPSKGAGEQGFIPWFSGKRTVNRLKEINTENDADANKAPPSGHNEAGFIPWFAGKRKV

tested peptides (round2):

WFSa: APPGNGEGEAGFIPWFS-amide

.

...

.....

________________________________________________________________________________________________________________________________________________________________________

--------

HLSamide

SNIamide

NTVamide

--------

>NVE1038

MKRRIHAILPVVALCVALSLANIDNGEMVERSGPAQPPLSHLSGKRASTWLRADPEQSIEMSRPLQPVDALEKGNSGKILQLDKVPMKMMIRQFGATPPSNIGRKRTNFRRYPPLNTVGKKREFCETREDIRNNAILD

tested peptides (round2):

HLSa: pyro-QPPLSHLS-amide

SNIa: TPPSNI-amide

NTVa: YPPLNTV-amide

.

...

.....

________________________________________________________________________________________________________________________________________________________________________

--------

IPIamide

IPFamide

--------

>NVE1473

MESFVLLAVLTYSLVSCTLVTPQSSKEEVATRWSKDKYDYLPLIPHGESSDESDAFLENILRNDKTTYREKKGRVVHKGKNPPQPPFRIPIGRKRSNRVVIHTGANPTMAPFRIPGGRKRSNRVVIHTGANPTMAPFRIPIGRKRSNRVVIHTGANPTMAPLRIPFGRKRSNGVVVHTNPDLSDLDGDTPNVNIERAQKEEC

tested peptides (round2):

pIPIa: pyro-QPPFRIPI-amide

IPIa: NPPQPPFRIPI-amide

IPFa: RSNRVVIHTGANPTMAPLRIPF-amide

.

...

.....

________________________________________________________________________________________________________________________________________________________________________

----------

QW peptide

----------

>NVE16920

MVKFEIFMGLALLLFVSADCLENLLQIKRDSSPPQWKRESTPPQWKRDSSPPQWKRDSSPPQWKRDSSPPQWKLDSSPPQWKRDSSPPQWKRDSSPPQWKRDSSPPQWKRDSSPPQWKQDLSPPQWKRESTPPQWKLESTPPQWKRDSSPPQWKRGIFH

tested peptides (round2):

QW1: TPPQW

QW2: DSSPPQW

QW3: SPPQWKLDSSPPQW

.

...

.....

________________________________________________________________________________________________________________________________________________________________________

-------

QIamide

-------

>NVE3868 (GQIa, Hayakawa 2022)

MQPLSVVIGVFVVLTITVLYTEGSPTGLDEREELYEAFMNARKAPLDIQSLFKLEDKRSAALPPRFGGQIGRRR

tested peptides (round2):

QIa: LPPRFGGQI-amide

.

...

.....

________________________________________________________________________________________________________________________________________________________________________

-----------

GWA peptide

-----------

>NVEC200_010517_1.1 (NVE6636)

MSRLVILLVFCLVLVSLALGQLITEGGEGINAGGACKRANGMACSDTTQGWEKVDMKRQVRISETTDPWVWEPKRQRLAKVGREQKEDEDNNLGGGGWFRRQAMQWGHKGWAKRQAQLNEGWKSVNDDKGWAKRQAQLNEGWKSVNDDKGWAKPQAQMNEGWKSVNDDKGWAKPHAQLNEGWKSVNDDKGWAKPQAQLNEGWKSANYDKGWAKRQAKLNKGWESANYDKGWAKRQAQLNDGWMHRQAAWRDEGKDKSGDKDLRFWGVAGRH

tested peptides (round2):

GWA1: pyro-QAMQWGHKGWA

GWA2: pyro-QAKLNKGWESANYDKGWA

.

...

.....

________________________________________________________________________________________________________________________________________________________________________

-----------

LRE peptide

-----------

>NVE4782

MKSLIQVLLLALVVIELAQCQLPSVSEVSENDVASLLRERRSLGYYIVSDWDECDCSTGWQSRQVSCAQPPCHFSRAPRPRRPCVC

tested peptides (round2):

LRE: pyro-QLPSVSEVSENDVASLLRE

.

...

.....

________________________________________________________________________________________________________________________________________________________________________

-----------

EFV peptide

-----------

>NVE8232

MKPYQKVLAGLLVVALAVQVVLSQETDGESLKKAEEEFVRRCAVYGCGLFDDFEDKRGLFTKLRLGGSKGGNRHYFKNRKYFRNNVVKRDANLNKLSVKDLDNE

tested peptides (round2):

EFV: pyro-QETDGESLKKAEEEFV

.

...

.....

________________________________________________________________________________________________________________________________________________________________________

-----------

GLWG peptide

-----------

>NVE21167

MLSLVALVTLALISPELASGSPFSQMRRSLLEEQCLPGVWGCKRGAPAALNEQRRTASEESDEQCLPGVWGCKKRTVIQERQCLPGVWGCKRGFDIPIQAKKKQKTPEECPPGLWGCKRGFLGGYYPETSPDYYQQGMPSEYSEYQQGYPSDYQNLASYYKRRLAIRMRRSVEKEDEQCLPGVWGCKKDKIEAKKSEDQCLPGVWGCKRDKIAKKQAEEQCPPGLWGCKKDNIEIEKTEETKKSEDQCLPGVWGCKKRSVTSN

tested peptide:

GLWG: QCPPGLWGC

GLWGcc: QCPPGLWGC (2-9 cysteine bridge)

.

...

.....

________________________________________________________________________________________________________________________________________________________________________

-----------

Phoenixin

-----------

>Nvec_comp12848_c0_seq2

>NVE21970 is missing N-terminus with signal peptide

MSSQVRSGIVIGGFVALTVAMMFPIAIYPKLFPEVYQDIQRDRRKGIKQEEIQPGNMKVWSDPFKKD*

tested peptide:

Pnx12: pyro-QPGNMKVWSDPF

Pxn14: EIQPGNMKVWSDPF

Pxn20: KGIKQEEIQPGNMKVWSDPF

.

...

.....

________________________________________________________________________________________________________________________________________________________________________
